# *In situ* expression of eukaryotic ice-binding proteins in microbial communities of Arctic and Antarctic sea ice

**DOI:** 10.1038/ismej.2015.43

**Published:** 2015-04-17

**Authors:** Christiane Uhlig, Fabian Kilpert, Stephan Frickenhaus, Jessica U Kegel, Andreas Krell, Thomas Mock, Klaus Valentin, Bánk Beszteri

**Affiliations:** 1 Polar Biological Oceanography, Alfred Wegener Institute Helmholtz Centre for Polar and Marine Research, Am Handelshafen 12, Bremerhaven, Germany; 2 Marine Geochemistry, Alfred Wegener Institute Helmholtz Centre for Polar and Marine Research, Am Handelshafen 12, Bremerhaven, Germany; 3 Computing Centre, Alfred Wegener Institute Helmholtz Centre for Polar and Marine Research, Am Handelshafen 12, Bremerhaven, Germany; 4 Hochschule Bremerhaven, Department of Technology, An der Karlstadt 8, Bremerhaven, Germany; 5 Ecological Chemistry, Alfred Wegener Institute Helmholtz Centre for Polar and Marine Research, Am Handelshafen 12, Bremerhaven, Germany; 6 School of Environmental Sciences, University of East Anglia, Norwich Research Park, Norwich NR4 7TJ, Norfolk, UK

## Abstract

Ice-binding proteins (IBPs) have been isolated from various sea-ice organisms. Their characterisation points to a crucial role in protecting the organisms in sub-zero environments. However, their *in situ* abundance and diversity in natural sea-ice microbial communities is largely unknown. In this study, we analysed the expression and phylogenetic diversity of eukaryotic IBP transcripts from microbial communities of Arctic and Antarctic sea ice. IBP transcripts were found in abundances similar to those of proteins involved in core cellular processes such as photosynthesis. Eighty-nine percent of the IBP transcripts grouped with known IBP sequences from diatoms, haptophytes and crustaceans, but the majority represented novel sequences not previously characterized in cultured organisms. The observed high eukaryotic IBP expression in natural eukaryotic sea ice communities underlines the essential role of IBPs for survival of many microorganisms in communities living under the extreme conditions of polar sea ice.

Sea ice constitutes one of the largest ecosystems in the world, accommodating a thriving community of microorganisms ranging from viruses to small metazoans (Thomas and Dieckmann, 2002; Mock and Thomas, 2005). Many of these organisms express ice-binding proteins (IBPs), which are regarded to be essential for the survival of sea ice species but are absent in temperate relatives (Janech *et al.*, 2006; Raymond and Kim, 2012; Raymond and Morgan-Kiss, 2013). Genes encoding this IBP class are found in distantly related organisms and were probably transferred via horizontal gene transfer inside and between the domains of bacteria and eukaryotes (Sorhannus, 2011; Raymond and Kim, 2012). The proteins change the microstructure of sea ice, thus helping to retain more liquid brine and making it more habitable to the microorganisms (Bayer-Giraldi *et al.*, 2011; Krembs *et al.*, 2011; Raymond and Kim, 2012). IBPs were shown to be highly expressed both at the transcript and protein level in two diatom species under laboratory conditions resembling sea ice (Bayer-Giraldi *et al.*, 2010, 2011). However, *in situ* IBP expression and diversity in natural sea ice communities is largely unknown. Here we tested whether IBPs are also highly expressed *in situ*, as can be expected based on their presumed critical role for survival in this habitat; and explored the phylogenetic diversity of IBP transcripts in natural sea ice communities.

We first analysed the abundance of eukaryotic IBP transcripts in five microbial sea ice communities sampled from both polar regions. Sanger sequenced metatranscriptomes (Supplementary Table 1) were established from RNA collected from Arctic sea ice (Kongsfjord, Svalbard: ARC, organism size 1.2–200 μm) and Antarctic sea ice (Dumont d'Urville Sea: ANT-A1, ANT-A2, organism size 0.2–50 μm; Weddell Sea: ANT-B1, ANT-B2, organism size >1.2 μm). A replicate of the samples ANT-B1/B2 was sequenced with Roche (Rotkreuz, Switzerland) 454 GS-FLX (NERC sequencing facility, Liverpool, UK) and GS-Titanium (Roche 454, Branford, CT, USA) techniques by Toseland *et al.* (2013). We included this dataset (454-ANT-B) in our analyses (Supplementary Table 1).

Transcripts encoding IBPs and, for comparison, reference genes responsible for core cellular functions (actin, light-harvesting proteins, protochlorophyllide reductase, oxygen-evolving enhancer protein 1 of photosystem II and 40 S ribosomal protein S4) were retrieved from the datasets based on sequence similarity (Supplementary Table 2). All metatranscriptomes contained type 1 IBP transcripts with abundances between 115 (ARC) and 1871 (ANT-B2) per 100 000 reads. These abundances were similar to or higher than those of the reference genes encoding core cellular functions (Figure 1). In comparison, we found no transcripts resembling type 2 IBPs (Raymond and Morgan-Kiss, 2013) and only 19 transcripts per 100 000 reads with similarity to bacterial ice nucleation proteins (Warren and Corotto, 1989) in one single sample (ARC).

Sample ANT-B2 differs considerably from the other metatranscriptomes containing an order of magnitude more IBP transcripts and higher transcript ratios of IBPs to photosynthetic reference genes (light-harvesting proteins, oxygen-evolving enhancer protein 1 of photosystem II). However, we are not able to explain these differences either by physical properties of the sea ice (temperature, salinity, ice type and thickness), filter size (Figure 2c) or by species composition (data not shown). Differences in the abundances of IBP transcripts, with 168 per 100 000 reads in 454-sequenced 454-ANT-B compared with 361 and 1871 per 100 000 reads in the Sanger-sequenced ANT-B1 and ANT-B2 (Figure 1) probably result from the different sequencing methods employed. We observed the same pattern for all reference genes (except actin) perhaps because our BLAST approach misses substantially more homologous sequences in the 454-dataset than in the Sanger reads. This might be caused by the low complexity of the 454-metatranscriptome assembly (Supplementary Material) or the shorter sequence length of the 454-isotigs compared with the Sanger singletons and contigs (Supplementary Table 3).

We next analysed the diversity of the environmental type 1 IBP transcripts by phylogenetic placement upon a backbone tree (Matsen *et al.*, 2010) (Supplementary Methods) constructed from 175 DUF3494 PfamA (http://pfam.xfam.org/) domains. Eighty-nine percent of the IBP transcripts from the metatranscriptomes were placed into two clades, which we refer to as ‘diatom' and ‘microalgae and copepod' clades (Figure 2). Unlike the mostly ‘microalgae and copepod'-dominated Antarctic samples, our Arctic sample (ARC) contained only sequences related to the ‘diatom' group (Figure 2b). This might point to the possibility that geographic isolation between both the polar regions has contributed to IBP evolution, but a broader bipolar survey will be necessary to confirm this. A *de novo* phylogeny calculated by using all environmental reads longer than 150 amino acids in addition to the PfamA reference set revealed a diversity of IBP sequences especially in the ‘microalgae and copepod' clade, which were not closely related to the terminal nodes (Supplementary Figure S2). This shows that an in-depth phylogenetic interpretation of environmental sequences will require a more comprehensive IBP inventory obtained from a broader diversity of sea ice organisms. Two horizontal gene transfer events have been documented within our individual named clades (Figure 2), as well as a transfer from fungi to diatoms (Sorhannus, 2011). However, the possibility of further horizontal gene transfer events in this gene family cannot be ruled out, limiting the possibilities of directly linking IBP phylogenetic positions to organismal taxonomy. Only 44% of the placements (accounting for 26% of the reads) were placed with a posterior probability above 75% (Supplementary Figure S1). Nevertheless, because of the grouping in our analysis (Figure 2a), a lower statistical threshold would not change the interpretation as long as alternative placements fall within the same group. This is the case for 92% of the placements, containing 99% of the reads.

Laboratory studies of IBP transcript (Bayer-Giraldi *et al.*, 2010; Kiko, 2010) and protein abundances (Bayer-Giraldi *et al.*, 2011) already indicated the importance of these proteins under freezing conditions. Our study provides the first evidence that type 1 IBP transcripts are highly abundant and diverse in natural microbial communities of the Arctic and Antarctic sea ice. The phylogenetic difference of the Arctic sample might be indicative of a geographic differentiation in the functionality and diversity of IBP genes, potentially reflecting either bipolar differences in how these microbial communities cope with conditions in sea ice, or the consequence of geographic isolation.

## Figures and Tables

**Figure 1:**
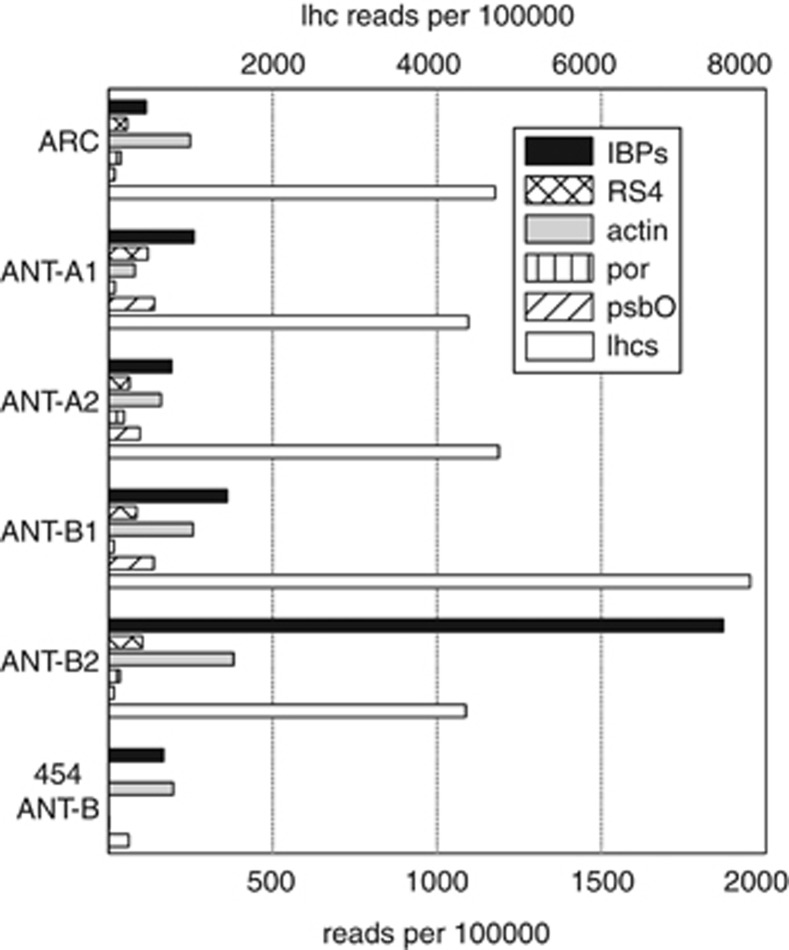
Abundances of IBP reads and reference genes of core cellular processes in the metatranscriptomes. Lhcs are shown on the secondary horizontal axis. lhcs, light-harvesting proteins; IBPs, ice-binding proteins; RS4, ribosomal protein S4; actin; por, protochlorophyllide reductase; psbO, oxygen-evolving enhancer protein 1 of photosystem II.

**Figure 2:**
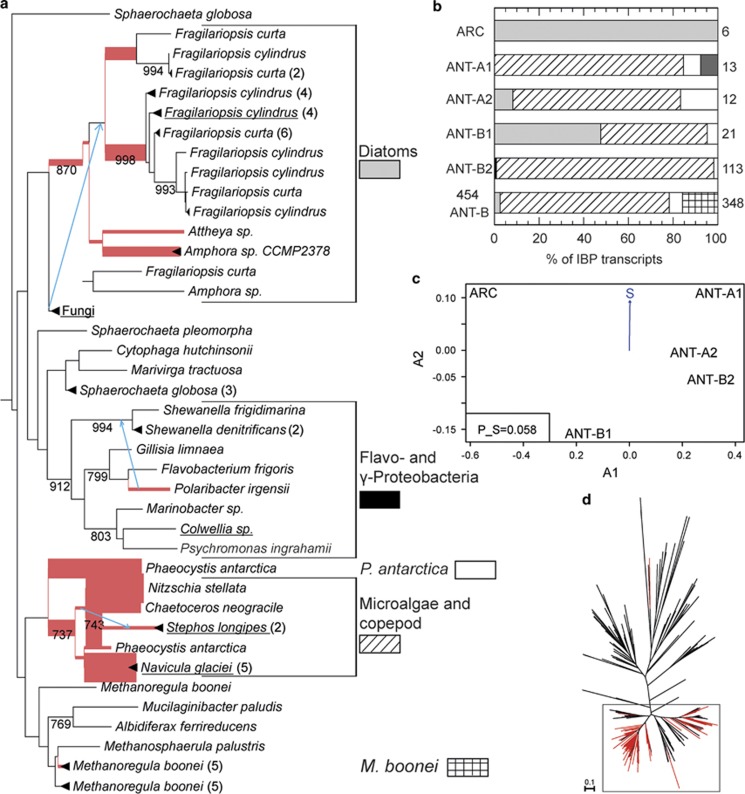
Placement of environmental IBP reads on a maximum likelihood tree of the PfamA DUF3494 domain calculated with PhyML (amino acid substitution LG and 1000 bootstraps) with pplacer. (**a**) Sub-tree containing 99% (71 out of 72) of the placements. Branches on which environmental IBPs were placed are coloured in red. The branch width indicates the number of placements on the respective branch. Only the highest placement is shown for each sequence. Clades containing sequences from a single species were collapsed and the number of sequences indicated in parenthesis. Bootstrap values larger than 700 are shown and sequences with known IBP-function according to Bayer-Giraldi *et al.* (2010) and Gwack *et al.* (2010) are underlined. Horizontal gene transfer events identified in Sorhannus (2011) are indicated by light blue arrows. (**b**) Assignment of the IBP transcripts to the groups indicated in the IBP tree in (a). Numbers at the right indicate the total number of IBP reads in the respective sample. Dark grey in ANT-A1 resembles sequences from *Candidatus* Aquiluna sp. and is located is outside the partial tree. (**c**) Principal coordinate analysis (PCO) of the Kantorovich–Rubinstein-distances of the pplacer analysis with environmental factors. Correlation with salinity is indicated as blue arrow and the *P*-value is given. (**d**) Complete unrooted backbone tree showing all placements are indicated by red branches. The square indicates the position of the subtree shown in (**a**).
