# Supplementary material for: In situ expression of eukaryotic ice-binding proteins in microbial communities of Arctic and Antarctic sea ice
Source: ISME J. 2015 Apr 17;9(11):2537–40. doi: 10.1038/ismej.2015.43 (PMC4611500; doi:10.1038/ismej.2015.43)
Supplement: Supplementary Information [file ismej201543x1.pdf]

Supplementary Material:

Supplementary Methods:

<u>Sampling</u>

Sea ice samples from the Southern Ocean were collected on cruise ANTXXIII-7 of RV Polarstern in the Weddell Sea in 2006 (sampling sites ANT-B1 and ANT-B2) and on the SIPEX cruise of RV Aurora Australis in the Dumont d´Urville Sea in 2007 (sampling sites ANT-A1 and ANT-A2) (Supplementary Table 1). The Arctic sample was retrieved from Kongsfjord, Svalbard, in spring 2009 (sampling site ARC) (Supplementary Table 1).

Ice samples were retrieved by collection of biomass rich ice pieces which were freshly broken by passage of the ship (ice fishing, for station ANT-B1) or by drilling (Kovacs drill 9 cm diameter, all other samples). The biomass rich sections of the ice cores (lower 1 - 10 cm) were taken, cut into slices, crushed and washed with cold sterile brine or sea water.

Organisms were collected on polycarbonate filters with pore size 1.2 µm for Kongsfjord (ARC) and Weddell Sea (ANT-B1, ANT-B2) samples and 0.2 µm for Dumont d´Urville (ANT-A1, ANT-A2) at 4 °C under vacuum not exceeding -200 mbar. Samples were prefiltered through a 50 µm mesh for ANT-A1 and ANT-A2 and a 200 µm mesh for ARC samples, to remove larger organisms. ANT-B1 and ANT-B2 were not prefiltered, but filters were checked visually for larger organisms and those were eventually removed. Filters were either treated with RNA*later*® (Applied Biosystems) (0.5 to 1.5 mL) and stored at -80 °C (ANT-A1, ANT-A2, ARC) or frozen dry and stored in liquid nitrogen (ANT-B1, ANT-B2, 454-ANT-B) until RNA extraction.

RNA preparation

For samples stored in RNA*later*®, RNA*later*® solution was removed by repeated centrifugation for 10 min at 10000 g to 16000 g at 4 °C. RNA was isolated using TRIreagent® (Sigma) according to the manufacturer's recommendations except the following modifications. TRIreagent® was heated to 60 °C before addition to the cell pellet, and glass beads (diameter 212 – 600 µm) were used to facilitate homogenization of the cells. Isopropanol precipitation was carried out at -20 °C and for samples stored in RNA*later*® 200 µL RNAse-free water was added to improve mixing of the isopropanol and the aqueous phase. DNA was digested using the RNAse-Free DNase Set (Qiagen) and RNA purified either by using the RNeasy kit (Qiagen) or by ammonium acetate precipitation.

Construction of cDNA libraries

Construction of cDNA libraries for all stations was accomplished by vertis Biotechnologie AG (Munich, Germany). First strand synthesis from total RNA was conducted using an oligo(dT)-linker primer and cDNA was further amplified with high fidelity polymerase. The plasmid vector pBS II sk+ was used for ligation of cDNA. For cloning, the ligations were electroporated into T1 Phage resistant TransforMax™ EC100™-T1R (Epicentre) electro-competent cells. Sanger sequencing of the complete cDNA libraries was performed by the Max-Planck genome centre Berlin/Cologne (Germany) using the M13 forward primer (TGT AAA ACG ACG GCC AGT).

Sanger reads are available under GenBank accession numbers JZ733060 to JZ761128.

454 raw data preparation

Samples for the 454 metatranscriptome of station ANT-B were treated as described in Toseland *et al.* (2013) and sequencing performed with Roche 454 GS-FLX and GS-Titanium techniques. Raw reads were assembled with Newbler (Roche, version 2.6) with default parameters for transcriptomic sequences. Only isotigs longer than 250bp were considered for analysis.

454 sequence data will soon be available at the NCBI Sequence Read Archive accession number SRR1752079.

Quantitative analysis with BLAST:

All datasets (contigs and singletons of the Sanger EST libraries and isotigs larger 250bp of the 454 metatranscriptome) were filtered for the presence of potential IBP sequences and sequences of reference genes responsible for core cellular functions (actin, fucoxanthin-chlorophyll-binding proteins (fcps), protochlorophyllide reductase (por), oxygen-evolving enhancer protein 1 of photosystem II (psbO) and 40S ribosomal protein S4 (RS4)) using local TBLASTN (BLAST 2.2.25) (Altschul *et al.*, 1997). Datasets were used as databases whereas sequences of the target genes listed in Supplementary Table 2 were used as queries, with a cutoff E-value of 0.1 in the blast run.

The resulting sequences for IBPs and reference genes were applied in an online BLASTX 2.2.29+ analysis against the refseq and swissprot databases, respectively, excluding models and uncultured samples. Queries that produced hits of the expected gene (e.g. IBP, RS4) with E-values $\leq 10^{-2}$ were kept for further analysis. For quantification the number of reads building one contig/isotig was taken into account (i.e. a contig with five reads was counted

as five and not one). Quantification of reads per 100,000 was based on unassembled reads for the EST databases and the number of assembled reads for the 454 metatranscriptome.

Phylogenetic analysis with pplacer:

Phylogeny of IBP transcripts was analyzed using the phylogenetic placement program pplacer v1.1alpha10 (Matsen *et al*., 2010). For the backbone tree the Pfam-alignment of domain DUF3494 (Pfam A) was downloaded from the Pfam database (http://pfam.xfam.org/). Six sequences with unknown taxonomy (from a mine drainage sample) were removed, resulting in an alignment of 175 domains with a length of 476bp. A maximum likelihood tree was calculated with PhyML 20120412 (Guindon and Gascuel, 2003) using default parameters (LG model for amino acid substitutions) and 1000 bootstraps. All translated IBP sequences retrieved from the metatranscriptomic libraries were aligned to a profile HMM calculated with HMMER 2.4 (Durbin *et al*., 1998) and placed into the reference tree with pplacer 1.1alpha10 (Matsen *et al*., 2010). Graphical output was generated using guppy and the trees were displayed and modified in Archaeopteryx (Han and Zmasek, 2009). Phylogenetic assignments were chosen according to the best placement (pplacer output parameter ML likelihood weight ratio: MLratio). Additionally, the posterior probability value of each placement was recorded (Supplementary Figure S1).

The placement was conducted with the singletons and contigs (EST libraries) as well as the isotigs (454 dataset) that were identified as IBPs in the BLAST analysis.

Principal coordinate analysis of phylogenetic diversity of IBP transcripts

Principal coordinate analysis (PCO) was calculated based on the phyloassigner placements using Kantorovich-Rubinstein-distances (Figure 2c) as implemented by Evans and Matsen (2012). PCO was performed with the R-package ade4 (Dray and Dufour, 2007). A fit of station data for salinity, temperature, ice thickness, daylight-hours as well as maximum and minimum filter size was performed for samples ANT-A1, ANT-A2, ANT-B1 (without temperature and salinity), ANT-B2 and ARC.

De novo analysis of phylogenetic diversity of the environmental IBP transcritps

We calculated a Profile-Alignment with HMMER 2.4 (Durbin *et al.*, 1998) from the DUF3494 PfamA alignment including the environmental IBP transcripts larger 150 amino acids. The resulting alignment was used to calculate a maximum likelihood tree with PhyML 20120412 (Guindon and Gascuel, 2003) using default parameters (LG model for amino acid substitutions) and 100 bootstraps. Environmental sequences smaller than 150 amino acids were placed onto this backbone tree using pplacer as described in material and methods.

Supplementary Table 1: List of samples used in this study including sampling date, position and physical properties of sea ice, as well as the size fractions collected by filtration. Data of stations ANT-A1 and ANT-A2 and J were adapted from Meiners *et al*. (2011) and for station ANT-B1 and ANT-B2 from Haas *et al*. (2009).

Station		Date	Latitude	Longitude	Ice thickness (m)	Salinity (range or mean)	Temp (°C)	Ice type at lower section	Age of ice	Size fraction (µm)	Identifier in Suppl. Fig. 1 and 2
**Antarctic**											
Weddell Sea	ANT-B1[1]	060923	60°07.150 S	47°54.550 W	1.46 (± 0.05)	n.d.	n.d.	n.d.	1st year ice	>1,2	awig5
	ANT-B2	061008	65°06.117 S	57°23.551 W	1.51 (± 0.57)	3.71	-1.9[2]	columnar	1st year ice	>1.2	awis5
	454-ANT-B	pool of parallel filters to samples ANT-B1 and ANT-B2									454(g3/s3)
Dumont d´Urville Sea	ANT-A1	070911	64°13.773 S	127°57.132 E	0.59 (0.52—0.70)	5.0 – 11.4	-5.7 (-9.7 to -2.3)	granular - columnar - granular	1st year pack ice	0.2-50	awiA4
	ANT-A2	071003	65°01.496 S	117°42.015 E	1.08 (1.05-1.09)	2.1 – 8.1	-4.5 (-6.8 to -2.0)	columnar	1st year pack ice	0.2-50	awiJ4
**Arctic**											
Kongsfjord	ARC	090504	78°57.550N	12°20.023 E	0.50	5.4 – 9.9	-2.01 (-2.1 to -1.6)	n. d.	1st year ice (spring melt)	1.2-200	awiKF1

[1] due to the sampling method (ice fishing) no information on physical ice properties is available for station ANT-B1, ice thickness and age were assumed to be similar to another station from 060923 and values adapted

[2] temperature at the ice water interface, where the sample was taken

Supplementary Table 2:

Table of the genes that were used for the blast search and full name for genes. Abbreviations for the genes, the full names, lengths of the sequences in amino acids, NCBI accession numbers as well as the originating organisms are given. For IBPs only part of the DUF3494 domain was used.

	Length [aa]	NCBI Acc. No.	organism
**type 1 IBPs**	140-155	ABH08428	*Colwellia sp*. SLW05
type 1 ice-binding		ACL00837	*Stephos longipes*
proteins (DUF3494		ACL00838	*Stephos longipes*
IBPs)		ACL27143	*Flammulina populicola*
		ACL27145	*Lentinula edodes* (shiitake mushroom)
		ACU09498	*Chaetoceros neogracile*
		YP_003095014	*Flavobacteriaceae bacterium* 3519-10
		ACU30806	*Leucosporidium sp.* AY30
		ACX36851	*Fragilariopsis cylindrus*
		ACX36853	*Fragilariopsis cylindrus*
		AEY75833	*Nitzschia stellata*
		AEY75834	*Amphora sp.* CCMP2378
		AEY75837	*Attheya sp.* CCMP212
		AEY75838	*Phaeocystis antarctica*
		BAD02891	*Typhula ishikariensis*
		AFK64811	*Pyramimonas gelidicola*
		AGC91914	*Chlamydomonas raudensis*
		AAZ76251	*Navicula glaciei*
		YP_943880	*Psychromonas ingrahamii* 37
**type 2 IBPs**	353-359	ABY64758	*Chlamydomonas sp. CCMP681*
Chlamydomonas sp		ABY64759	*Chlamydomonas sp. CCMP681*
CCMP 681 ice binding		ABY64761	*Chlamydomonas sp. CCMP681*
proteins		ABY64760	*Chlamydomonas sp. CCMP681*
**por**	420-440	XP_002294544	*Thalassiosira pseudonana* CCMP1335
protochlorophyllide		XP_002179689	*Phaeodactylum tricornutum* CCAP 1055/1
reductase		XP_005853690	*Nannochloropsis gaditana* CCMP526
		XP_005784495	*Emiliania huxleyi* CCMP1516
**psbO**	305-314	XP_002180309	*Phaeodactylum tricornutum* CCAP 1055/1
oxygen-evolving		XP_002291225	*Thalassiosira pseudonana* CCMP1335
enhancer protein 1 of		XP_005855446	*Nannochloropsis gaditana* CCMP526
photosystem II		XP_005761454	*Emiliania huxleyi* CCMP1516

Supplementary Table 2 continuing:

	Length [aa]	NCBI Acc.	organism
**RS4**	260	XP_002288080	*Thalassiosira pseudonana* CCMP1335
40S ribosomal protein		XP_002177120	*Phaeodactylum tricornutum* CCAP 1055/1
S4		XP_001691218	*Chlamydomonas reinhardtii*
**fcps**	205-400	XP_002292153	*Thalassiosira pseudonana* CCMP1335
fucoxanthin chl a/c		XP_002292353	*Thalassiosira pseudonana* CCMP1335
light-harvesting protein		XP_002289005	*Thalassiosira pseudonana* CCMP1335
		XP_002288517	*Thalassiosira pseudonana* CCMP1335
		XP_005767752	*Emiliania huxleyi* CCMP1516
		XP_005786132	*Emiliania huxleyi* CCMP1516
		XP_005778279	*Emiliania huxleyi* CCMP1516
		XP_005778485	*Emiliania huxleyi* CCMP1516
		XP_001698519	*Chlamydomonas reinhardtii*
		XP_001693987	*Chlamydomonas reinhardtii*
		XP_001700243	*Chlamydomonas reinhardtii*
		XP_001694115	*Chlamydomonas reinhardtii*
		XP_001701405	*Chlamydomonas reinhardtii*
		XP_001698542	*Chlamydomonas reinhardtii*
		XP_001701405	*Chlamydomonas reinhardtii*
		XP_001698542	*Chlamydomonas reinhardtii*
		XP_001694115	*Chlamydomonas reinhardtii*
		XP_001695467	*Chlamydomonas reinhardtii*
		XP_001695344	*Chlamydomonas reinhardtii*
		XP_001695353	*Chlamydomonas reinhardtii*
		XP_001703699	*Chlamydomonas reinhardtii*
		XP_001697526	*Chlamydomonas reinhardtii*
		XP_001695466	*Chlamydomonas reinhardtii*
		XP_001691959	*Chlamydomonas reinhardtii*
		XP_001696202	*Chlamydomonas reinhardtii*
		XP_001692548	*Chlamydomonas reinhardtii*
		XP_001699932	*Chlamydomonas reinhardtii*
		XP_002958754	*Volvox carteri f. nagariensis*
		NP_173034	*Arabidopsis thaliana* (thale cress)
**actin**	280-380	XP_002294917	*Thalassiosira pseudonana* CCMP1335
		AFO84294	*Ditylum brightwellii*
		ABC54738	*Skeletonema costatum*
		XP_002183424	*Phaeodactylum tricornutum* CCAP 1055/1
		ABQ45363	*Nitzschia closterium f. minutissima*
		AAO92429	*Phytophthora brassicae*

Supplementary Table 2 continuing:

	Length [aa]	NCBI Acc.	organism
**INP**	1200-2145	P16239	*Pantoea agglomerans*
Ice nucleation proteins		P06620	*Pseudomonas syringae pv. syringae*
		AAK70465	*Pantoea ananatis*
		P20469	*Pantoea ananatis*
		BAK13807	*Pantoea ananatis AJ13355*
		ACB59244	*Pseudomonas borealis*
		CBI99147	*Pseudomonas syringae*
		YP_001033817	*Rhodobacter sphaeroides 2.4.1*
		YP_003576802	*Rhodobacter capsulatus SB 1003*
		YP_419798	*Magnetospirillum magneticum AMB-1*

Supplementary Table 3:

Read and contig/isotig length (in basepairs) of Sanger and 454 data

Sanger sequenced datasets			
	**number of reads**	**average read length**	**average contig length**
**ARC**	5228	654	772
**ANT-A1**	5002	665	829
**ANT-A2**	6254	699	813
**ANT-B1**	5812	584	715
**ANT-B2**	5773	580	767
454 sequenced dataset			
	**number of aligned reads**	**average read length**	**average isotig length**
**454-ANT-B[1] total**	206983	179	391
**454-ANT-B >250bp**			495

[1]454-ANT-B is a sequence pool obtained from parallel filters of samples ANT-B1 and ANT-B2 (Toseland *et al.*, 2013)

Supplementary Newbler assembly statistics of sample 454-ANT-B:

Input		
Number of reads	391614	
Number of bases	65831723	
Number of reads trimmed	290013	74.1%
Number of bases trimmed	51875003	78.8%
Consensus results		
Number of reads assembled	157097	54.2%
Number partial	49886	17.2%
Number singleton	31652	10.9%
Number repeat	3468	1.2%
Number outlier	1063	0.4%
Number too short	46847	16.2%
Isogroup Metrics		
Number of isogroups	1725	
Average contig count	1.0	
Largest contig count	1	
Number with one contig	1725	
Average isotig count	1.0	
Largest isotig count	1	
Number with one isotig	1725	
Isotig Metrics		
Number of Isotigs	1725	
Average contig count	1.0	
Largest contig count	1	
Number with one contig	1725	
Number of bases	675898	
Average isotig size	391	
N50 isotig size	451	
Largest isotig	2329	
Large Contig Metrics		
Number of contigs	347	
Number of bases	267138	
Average contig size	769	
N50 contig size	772	
Largest contig size	2329	
Q40 plus bases	253142	94.76%
All Contig Metrics		
Number of contigs	1725	
Number of bases	675898	
Average contig size	392	

Caption to Supplementary Figure S1:

Placements of all environmental IBPs into the backbone tree are shown on the red branches. Sequence names carry a sample identifier (awiKF1: ARC, awiA4: ANT-A1, awiJ4: ANT-A2, awig5: ANT-B1, awis5: ANT-B2, 454(g3/s3): 454-ANT-B). Numbers in brackets show the maximum likelihood weight ratio (like weight ratio) and the posterior probability (PP) of a placement on an edge from the pplacer analysis. Sequences marked in yellow are placed with high robustness (PP >75%). Sequences marked in blue have a low PP support and alternative placements of the respective read were found in a different group of the backbone tree. Five reads are alternating between the "Microalgae and crustacean" clade and a directly neighbouring *Phaeocystis antarctica* sequence. One read from the "Diatom" clade has the alternative placement in a group of closely related fungal sequences. The latter could be explained by horizontal gene transfer of IBP sequences from a basidiomycete to *Fragilariopsis sp.* (Sorhannus, 2011).

Caption to Supplementary Figure S2:

De novo analysis of the phylogenetic diversity of the environmental IBP transcripts. The backbone tree (PhyML 20120412, LG model for amino acid substitutions, 100 bootstraps) was constructed from the DUF3494 PfamA alignment and the environmental IBP transcripts longer than 150 amino acids (HMMER2.4 Profile-Alignment). Transcripts shorter than 150 amino acids were placed with pplacer and are shown on red branches. Environmental sequences are shown in blue and carry a sample identifier in the name (awiKF1: ARC, awiA4: ANT-A1, awiJ4: ANT-A2, awig5: ANT-B1, awis5: ANT-B2, 454(g3/s3): 454-ANT-B). A previously unknown diversity of the environmental IBPs emerges in the "Microalgae and copepod" clade which is shadowed in grey.

Supplementary References:

Altschul SF, Madden TL, Schäffer AA, Zhang J, Zhang Z, Miller W, Lipman DJ. (1997). Gapped BLAST and PSI-BLAST: a new generation of protein database search programs. Nucleic Acids Res 25:3389–3402. doi: 10.1093/nar/25.17.3389.

Durbin R, Eddy S, Krogh A, Mitchison G. (1998). Biological sequence analysis: probabilistic models of proteins and nucleic acids. Cambridge University Press.

Dray S, Dufour A (2007). The ade4 package: implementing the duality diagram for ecologists. Journal of Statistical Software 22:1–20.

Evans SN, Matsen FA (2012). The phylogenetic Kantorovich-Rubinstein metric for environmental sequence samples. Journal of the Royal Statistical Society: Series B (Statistical Methodology) 74:569–592. doi: 10.1111/j.1467-9868.2011.01018.x.

Guindon S, Gascuel O. (2003). A simple, fast, and accurate algorithm to estimate large phylogenies by maximum likelihood. Systematic Biology 52:696–704. doi: 10.1080/10635150390235520.

Haas C, Friedrich A, Li NM Zijun, Pfaffling A, Toyota T. (2009). Regional variability of sea ice properties and thickness in the northwestern Weddell Sea obtained by in-situ and satellite measurements. In: Lemke P. (ed.) The expedition of the research vessel “Polarstern” to the Antarctic in 2006 (ANT-XXIII/7), vol. 586 of Berichte zur Polar- und Meeresforschung. Alfred-Wegener-Institut für Polar- und Meeresforschung, Bremerhaven, p. 36–74.

Han M, Zmasek C. (2009). phyloXML: XML for evolutionary biology and comparative genomics. BMC Bioinformatics 10:356. doi: 10.1186/1471-2105-10-356.

Matsen F, Kodner R, Armbrust EV. (2010). pplacer: linear time maximum-likelihood and bayesian phylogenetic placement of sequences onto a fixed reference tree. BMC Bioinformatics 11:538. doi: 10.1186/1471-2105-11-538.

Meiners KM, Norman L, Granskog MA, Krell A, Heil P, Thomas DN. (2011). Physico-ecobiogeochemistry of East Antarctic pack ice during the winter-spring transition. Deep Sea Research Part II: Topical Studies in Oceanography 58:1172 – 1181. doi: DOI: 10.1016/j.dsr2.2010.10.033. Antarctic Sea Ice Research during the International Polar Year 2007-2009.

Sorhannus U. (2011). Evolution of antifreeze protein genes in the diatom genus *Fragilariopsis*: Evidence for horizontal gene transfer, gene duplication and episodic diversifying selection. Evolutionary Bioinformatics 7:279–289. doi: 10.4137/EBO.S8321.

Toseland A, Daines SJ, Clark JR, Kirkham A, Strauss J, Uhlig C, et al. (2013). The impact of temperature on marine phytoplankton resource allocation and metabolism. Nature Clim Change 3:979–984. doi: 10.1038/nclimate1989.
