# Supplementary figures and images for: In situ expression of eukaryotic ice-binding proteins in microbial communities of Arctic and Antarctic sea ice
Source: ISME J. 2015 Apr 17;9(11):2537–40. doi: 10.1038/ismej.2015.43 (PMC4611500; doi:10.1038/ismej.2015.43)

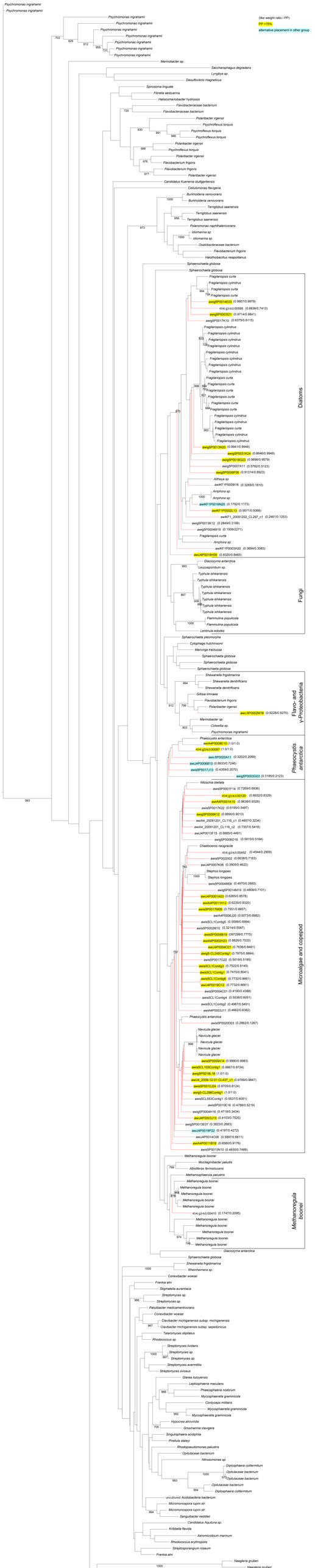

Supplement: Supplementary Figure S1 [file ismej201543x2.pdf]
