# Supplementary material for: In situ expression of eukaryotic ice-binding proteins in microbial communities of Arctic and Antarctic sea ice
Source: ISME J. 2015 Apr 17;9(11):2537–40. doi: 10.1038/ismej.2015.43 (PMC4611500; doi:10.1038/ismej.2015.43)
Supplement: Supplementary Figure S2 [file ismej201543x3.pdf]

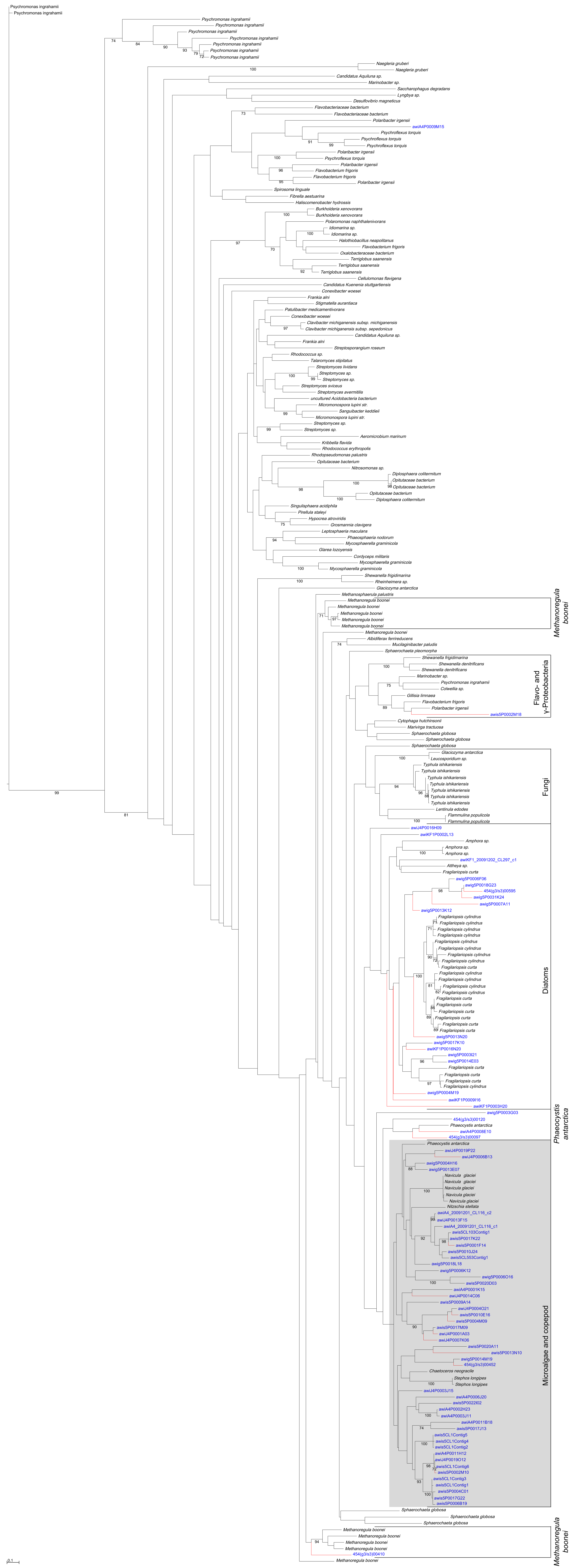

Psychromonas ingrahamii
Psychromonas ingrahamii
Psychromonas ingrahamii
Psychromonas ingrahamii
Psychromonas ingrahamii
Psychromonas ingrahamii
Psychromonas ingrahamii
Psychromonas ingrahamii
Psychromonas ingrahamii
Naegleria gruberi
Naegleria gruberi
Candidatus Aquiluna sp.
Marinobacter sp.
Saccharophagus degradans
Lyngbya sp.
Desulfovibrio magneticus
Flavobacteriaceae bacterium
Flavobacteriaceae bacterium
Polaribacter irgensii
awiA4P0009M15
Psychroflexus torquis
Psychroflexus torquis
Psychroflexus torquis
Polaribacter irgensii
Psychroflexus torquis
Polaribacter irgensii
Flavobacterium frigoris
Flavobacterium frigoris
Polaribacter irgensii
Spirosoma linguale
Fibrella aestuarina
Haliscomenobacter hydrossis
Burkholderia xenovorans
Burkholderia xenovorans
Polaromonas naphthalenivorans
Idiomarina sp.
Idiomarina sp.
Halothiobacillus neapolitanus
Flavobacterium frigoris
Oxalobacteraceae bacterium
Terriglobus saanensis
Terriglobus saanensis
Terriglobus saanensis
Cellulomonas flavigena
Candidatus Kuenenia stuttgartiensis
Conexibacter woesei
Frankia alni
Stigmatella aurantiaca
Patulibacter medicamentivorans
Conexibacter woesei
Clavibacter michiganensis subsp. michiganensis
Clavibacter michiganensis subsp. sepedonicus
Candidatus Aquiluna sp.
Frankia alni
Streptosporangium roseum
Rhodococcus sp.
Talaromyces stipitatus
Streptomyces lividans
Streptomyces sp.
Streptomyces sp.
Streptomyces sviceus
Streptomyces avermitilis
uncultured Acidobacteria bacterium
Micromonospora lupini str.
Sanguibacter keddieii
Micromonospora lupini str.
Streptomyces sp.
Streptomyces sp.
Aeromicrobium marinum
Kribbella flavida
Rhodococcus erythropolis
Rhodopseudomonas palustris
Opitutaceae bacterium
Nitrosomonas sp.
Diplosphaera colitermitum
Opitutaceae bacterium
Opitutaceae bacterium
Opitutaceae bacterium
Diplosphaera colitermitum
Singulisphaera acidiphila
Pirellula staleyi
Hypocrea atroviridis
Grosmannia clavigera
Leptosphaeria maculans
Phaeosphaeria nodorum
Mycosphaerella graminicola
Glarea lozoyensis
Cordyceps militaris
Mycosphaerella graminicola
Mycosphaerella graminicola
Shewanella frigidimarina
Rheinheimera sp.
Glaciozyma antarctica
Methanosphaerula palustris
Methanoregula boonei
Methanoregula boonei
Methanoregula boonei
Methanoregula boonei
Methanoregula boonei
Methanoregula boonei
Methanoregula boonei
Albidiferax ferrireducens
Mucilaginibacter paludis
Sphaerochaeta pleomorpha
Shewanella frigidimarina
Shewanella denitrificans
Shewanella denitrificans
Marinobacter sp.
Psychromonas ingrahamii
Colwellia sp.
Gillisia limnaea
Flavobacterium frigoris
Polaribacter irgensii
awis5P0002M18
Flavo- and γ-Proteobacteria
Cytophaga hutchinsonii
Marivirga tractuosa
Sphaerochaeta globosa
Sphaerochaeta globosa
Sphaerochaeta globosa
Glaciozyma antarctica
Leucosporidium sp.
Typhula ishikariensis
Typhula ishikariensis
Typhula ishikariensis
Typhula ishikariensis
Typhula ishikariensis
Typhula ishikariensis
Typhula ishikariensis
Lentinula edodes
Flammulina populicola
Flammulina populicola
Fungi
awiJ4P0016H09
awiKF1P0002L13
Amphora sp.
Amphora sp.
Amphora sp.
awiKF1_20091202_CL297_c1
Attheya sp.
Fragilariopsis curta
awig5P0006F06
awig5P0018G23
454(g3/s3)00595
awig5P0031K24
awig5P0007A11
awig5P0013K12
Fragilariopsis cylindrus
Fragilariopsis cylindrus
Fragilariopsis cylindrus
Fragilariopsis cylindrus
Fragilariopsis cylindrus
Fragilariopsis cylindrus
Fragilariopsis cylindrus
Fragilariopsis cylindrus
Fragilariopsis curta
Fragilariopsis cylindrus
Fragilariopsis cylindrus
Fragilariopsis cylindrus
Fragilariopsis cylindrus
Fragilariopsis curta
Fragilariopsis curta
Fragilariopsis curta
Fragilariopsis curta
Fragilariopsis curta
Fragilariopsis curta
awig5P0013N20
awig5P0017K10
awiKF1P0016N20
awig5P0003I21
awig5P0014E03
Fragilariopsis curta
Fragilariopsis curta
Fragilariopsis curta
Fragilariopsis cylindrus
awig5P0004M19
awiKF1P0009I16
awiKF1P0003H20
Diatoms
awig5P0003G03
454(g3/s3)00120
Phaeocystis antarctica
awiA4P0008E10
454(g3/s3)00097
Phaeocystis antarctica
Phaeocystis antarctica
awiJ4P0019P22
awiJ4P0006B13
awig5P0004H16
awig5P0013E07
Navicula glaciei
Navicula glaciei
Navicula glaciei
Navicula glaciei
Navicula glaciei
Nitzschia stellata
awiA4_20091201_CL116_c2
awiJ4P0013F15
awiA4_20091201_CL116_c1
awis5CL103Contig1
awis5P0017K22
awis5P0001F14
awis5P0010J24
awis5CL553Contig1
awig5P0018L18
awig5P0006K12
awig5P0006O16
awis5P0020D03
awiA4P0001K15
awiJ4P0014C06
awis5P0009A14
awiJ4P0004O21
awis5P0010E16
awis5P0004M09
awis5P0017M09
awiJ4P0001A03
awiJ4P0007K06
awis5P0020A11
awis5P0013N10
awig5P0014M19
454(g3/s3)00452
Chaetoceros neogracile
Stephos longipes
Stephos longipes
awiJ4P0003J15
awiA4P0006J20
awis5P0022I02
awiA4P0002H23
awiA4P0003J11
awiA4P0011B18
awis5P0017J13
awis5CL1Contig5
awis5CL1Contig4
awis5CL1Contig2
awiA4P0011H12
awiJ4P0019O12
awis5CL1Contig6
awis5P0002M10
awis5CL1Contig3
awis5CL1Contig1
awis5P0004C01
awis5P0017G22
awis5P0006B19
Microalgae and copepod
Sphaerochaeta globosa
Sphaerochaeta globosa
Sphaerochaeta globosa
Methanoregula boonei
Methanoregula boonei
Methanoregula boonei
Methanoregula boonei
454(g3/s3)00410
Methanoregula boonei
Methanoregula boonei
